# Abattoir-based study of *Salmonella* prevalence in pigs at slaughter in Great Britain

**DOI:** 10.1017/S0950268821001631

**Published:** 2021-09-02

**Authors:** F. Martelli, C. Oastler, A. Barker, G. Jackson, R. P. Smith, R. Davies

**Affiliations:** Animal and Plant Health Agency, Woodham Lane, New Haw, Addlestone, KT15 3NB, UK

**Keywords:** *Salmonella* prevalence, pigs, abattoir

## Abstract

Consumption of pork and pork products can be associated with outbreaks of human salmonellosis. *Salmonella* infection is usually subclinical in pigs, and farm-based control measures are challenging to implement. To obtain data on *Salmonella* prevalence, samples can be collected from pigs during the slaughter process. Here we report the results of a Great Britain (GB) based abattoir survey conducted by sampling caecal contents from pigs in nine British pig abattoirs during 2019. Samples were collected according to a randomised stratified scheme, and pigs originating from 286 GB farms were included in this survey. *Salmonella* was isolated from 112 pig caecal samples; a prevalence of 32.2% [95% confidence interval (CI) 27.4–37.4]. Twelve different *Salmonella* serovars were isolated, with the most common serovars being *S*. 4,[5],12:i:-, a monophasic variant of *Salmonella* Typhimurium (36.6% of *Salmonella-*positive samples), followed by *S.* Derby (25.9% of *Salmonella-*positive samples). There was no significant difference compared to the estimate of overall prevalence (30.5% (95% CI 26.5–34.6)) obtained in the last abattoir survey conducted in the UK (2013). Abattoir-based control measures are often effective in the reduction of *Salmonella* contamination of carcasses entering the food chain. In this study, the effect of abattoir hygiene practices on the prevalence of *Salmonella* on carcasses was not assessed. Continuing *Salmonella* surveillance at slaughter is recommended to assess effect of farm-based and abattoir-based interventions and to monitor potential public health risk associated with consumption of *Salmonella-*contaminated pork products.

## Introduction

In the European Union (EU) in 2019, 90 105 human salmonellosis cases were reported, of which 9718 were reported from the United Kingdom (UK) [1]. In 2018, of the 269 EU foodborne outbreaks with strong evidence for their source, 16 (5.4%) were linked to pig meat [2]. *Salmonella* prevalence in UK pigs is assessed at slaughter through the collection and testing of carcass swabs according to Commission Regulation EC No. 2073/2005 (as amended March 2014). In 2019, of the 3785 carcasses tested in the UK, 1.72% [95% confidence interval (CI) 1.33–2.18%] according to Commission Regulation EC No. 2073/2005 (as amended March 2014) were *Salmonella* positive [1]. This was a reduction compared to 2018, when 2.87% (95% CI 2.36–3.44%) of the 3839 carcasses tested in the UK were positive for *Salmonella* [2]. These samples were taken by food business operators and tested on a commercial basis in a range of private laboratories.

Additional abattoir surveys to assess *Salmonella* prevalence are conducted at regular intervals, and samples are tested at the national reference laboratory for *Salmonella*. The last published survey assessing the *Salmonella* prevalence of UK pigs at slaughter was conducted in 2013 by Powell *et al*. [3] and this reported *Salmonella* from 9.6% (95% CI 7.3–11.9) of tested carcass swab samples. This was a significant reduction compared to 15% (95% CI 12–18%) *Salmonella-*positive carcass swabs reported by Marier *et al*. [4] in the 2006–2007 *Salmonella* survey of slaughter pigs. This decrease in *Salmonella* contamination of carcasses was attributed to improvements made to abattoir hygiene control standards. However, the percentage of *Salmonella*-positive results obtained from caecal contents of the same pigs increased from 22% (95% CI 19–26%) of caecal contents in 2006–2007 to 30.5% (95% CI 26.5–34.6) in 2013 [3, 4].

The lower prevalence in carcass swabs is likely to be related to the effectiveness of hygienic measures at slaughter, which are generally regarded as more practical for reducing *Salmonella* on pig carcasses than interventions at primary production level [5]. Hygiene measures at abattoir level (such as scalding, singeing and blast chilling) reduce levels of surface contamination at slaughter, where environmental contamination before and after stunning is likely to be high [6].

The prevalence of *Salmonella-*infected pigs entering the abattoir has a direct impact on the levels of environmental contamination at slaughter [7]. Control measures applied on farm to reduce the intestinal carriage of *Salmonella* in pigs going to slaughter are therefore helpful in reducing the number of contaminated carcasses entering the food chain [8], but are difficult and expensive to implement [9].

Pigs arriving at slaughter normally have higher *Salmonella* prevalence than they had at the farm of origin. This is associated to the fact that carrier pigs might restart shedding after being exposed to stressful events (such as transport) or to the fact that new contaminations and infections might occur during transit or at the abattoir (e.g. in trucks or lairage) [10].

During 2019, as part of the harmonised monitoring of antimicrobial resistance (AMR) in zoonotic and commensal bacteria (Commission Decision 2013/652/EC), caecal samples were collected from UK pigs at slaughter, and tested for the presence of indicator *Escherichia coli* and *E. coli* resistant to selected antimicrobials.

The samples collected from British slaughterhouses were also tested for *Salmonella* in order to determine the *Salmonella* prevalence in pigs slaughtered in Great Britain (GB). This study aimed to estimate the prevalence of *Salmonella* infection in GB finisher pigs at slaughter and detect any change in prevalence from the 30.5% estimate obtained in 2013.

## Methods

### Sample collection

Pig caecal samples were collected as part of the harmonised monitoring of AMR in zoonotic and commensal bacteria (Commission Decision 2013/652/EC) carried out for pigs during 2019. Using a randomised stratified scheme, caecal samples, across 9 GB abattoirs, were collected by abattoir personnel or staff from the Food Standards Agency. The abattoirs were chosen according to their sampling throughput (at least 60% of the national production, starting with the largest abattoir). The sampling schedule was randomised and weighted according to throughput, as well as stratified by month for the year of the survey. From each chosen fattening herd, at least 11 g of caecal contents was collected from one randomly selected pig. Caecal contents were chilled and transported at 2–8 °C to the Animal and Plant Health Agency (APHA) laboratory for *Salmonella* determination within 96 h of collection.

### Bacteriological analysis

Caecal contents were tested for the presence of *Salmonella* using a modified version of ISO6579:2017. Ten grams of each caecal contents sample was added to 225 ml Buffered Peptone Water (BPW; Merck, Feltham, UK), and incubated at 37 ± 1 °C for 16–20 h. Following incubation, 0.1 ml of the enriched broth was inoculated onto modified semi-solid Rappaport-Vassiliadis agar (MSRV; Mast, Bootle, UK, with addition of 1 mg/ml of novobiocin; Sigma, Sigma-Aldrich Company Ltd, Dorset, UK) and incubated at 41.5 ± 1 °C for 24 ± 3 h. Growth on MSRV agar was collected using a 1 μl loop from the edge of the growth zone and sub-cultured onto three selective agars: Rambach agar (Merck, Feltham, UK); Brilliant Green Agar (BGA (modified); Oxoid, Basingstoke, UK, with addition of 1 mg/ml of novobiocin; Sigma, Sigma-Aldrich Company Ltd, Dorset, UK); and Xylose Lysine Desoxycholate agar (XLD; BD Difco; Becton, Dickinson and company, Berkshire, UK), and incubated at 37 ± 1 °C for 24 ± 3 h. MSRV plates were incubated for a further 24 ± 3 h at 41.5 ± 1 °C. Any MSRV plates which were initially negative for *Salmonella* growth, but showed positive growth after 48 h incubation were sub-cultured again onto Rambach, XLD and BGA agars. Suspect *Salmonella* isolates were confirmed by full serotyping according to the White-Kauffmann-LeMinor Scheme [11, 12]. A selection of the *S.* Typhimurium and monophasic *S.* Typhimurium (mST) strains were also phage typed [13].

### Statistical analysis

The sample size was sufficient to allow a *Salmonella* prevalence amongst slaughter pigs of 30% to be estimated with 95% confidence and 6% precision, and a 25–30% change in prevalence (e.g. a change of ±8% from a prevalence of 30%) would be detected with 95% confidence (Ausvet Epitools). *Salmonella* prevalence values were adjusted to account for multiple caecal samples collected from pigs originating from the same farm using the svy command in STATA (STATA16, StataCorp, College Station, USA). Chi-squared tests were used to assess whether there was statistical difference between the current prevalence estimate and the previous estimate in 2013, and comparing the *Salmonella* prevalence from the nine GB abattoirs and between the months that samples were collected.

## Results

A total of 348 pig caecal samples were tested for the presence of *Salmonella*. The pigs originated from 286 GB farms, with the majority of pigs originating from farms in England (94.3%) followed by Scotland (4.3%) and Wales (1.4%) (this reflects the distribution of pig herds in the different GB regions). *Salmonella* was isolated from 112 pig caecal samples; a prevalence of 32.2% (95% CI 27.4–37.4). This was not significantly different (*χ*^2^ *P*-value = 0.646) to the previous prevalence estimate of 30.5% (95% CI 26.5–34.6) from the 2013 study (Powell *et al*. [3]).

There was no significant difference in *Salmonella* isolation from the caecal samples collected at each of the nine abattoirs (*χ*^2^, *P*-value = 0.170) (Table 1).

**Table 1.** Number of samples collected at each of the nine abattoirs, and proportion of positive *Salmonella* samples per abattoir

Abattoir	*Salmonella* positive	No. samples	% Positive
1	12	40	30.0
2	1	11	9.1
3	14	63	22.2
4	13	36	36.1
5	16	41	39.0
6	27	59	45.8
7	11	36	30.6
8	4	16	25.0
9	14	44	31.8
Total	112	346	32.4

This table includes only 346 samples, as two samples could not be linked to an abattoir.

Twelve different *Salmonella* serovars were isolated from the caecal samples, with the most commonly isolated serovars being *S*. 4,[5],12:i:-, a monophasic variant of *S.* Typhimurium (36.6% of *Salmonella* positive samples), followed by *S.* Derby (25.9% of *Salmonella-*positive samples). *S.* Typhimurium and monophasic variant *S*. 4,[5],12:i:- accounted for 41.1% of *Salmonella* isolated (*S*. 4,[5],12:i:- 36.6% *S.* Typhimurium 4.5%). *S.* Ohio and *S.* Kedougou were only isolated from a single caecal sample each (Table 2).

**Table 2.** Serotype distribution in *Salmonella*-positive caecal samples from GB pigs at slaughter, and comparison of prevalence of same serovar in 2013 UK survey [3] (caecal samples only *n* = 619; total positive samples = 189)

Salmonella Serotype	No. positive samples (%)	% of all positive 2013 survey's caecal samples [3]
*S.* 4,[5]12:i:-	41 (36.6)	16.9
*S.* Derby	29 (25.9)	14.3
*S.* Newport	8 (7.1)	ND
*S.* Panama	8 (7.1)	3.2
*S.* Rissen	8 (7.1)	1.6
*S.* Typhimurium	5 (4.5)	19.0
*S.* Bovismorbificans	5 (4.5)	10.6
*S.* London	2 (1.8)	2.6
*S.* Mbandaka	2 (1.8)	0.5
*S.* Reading	2 (1.8)	4.2
*S.* Kedougou	1 (0.9)	3.2
*S.* Ohio	1 (0.9)	0.5

ND, not detected.

**Fig. 1.**
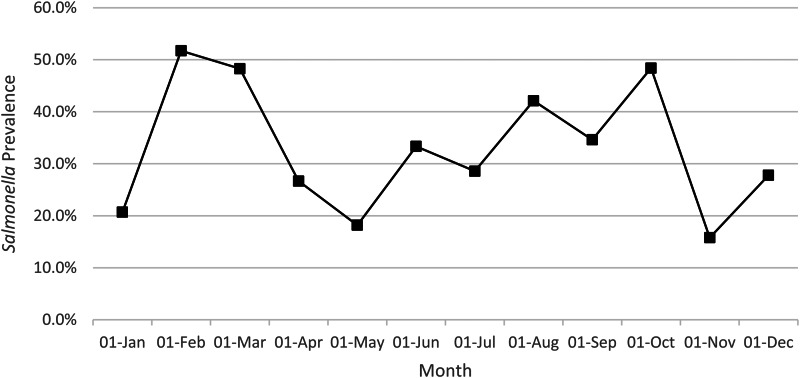
Percentage of *Salmonella*-positive caecal samples by month of sampling (2019).

Caecal samples were collected over a 12 month period, with between 19 and 38 caecal samples tested each month. Month to month variations in *Salmonella*-positive samples were noted, with peaks in *Salmonella* isolated from caecal contents in February to March and October (Fig. 1). No significant difference in prevalence was detected when comparing the results from the four seasons. At the monthly level, when each individual month was compared to a summary of the remaining months, February, March and October all had significantly higher prevalence (*χ*^2^ *P*-value <0.001, 0.002 and 0.001 respectively).

## Discussion

The 2019 prevalence of *Salmonella* in the GB finisher pig population at slaughter was estimated to be 32.2%, with some monthly variations in prevalence observed. There was no significant difference from the estimate obtained by Powell *et al*. [3], suggesting that *Salmonella* prevalence in pig caecal contents at abattoir level has not significantly changed in the period 2013–2019. Carriage of *Salmonella* in pigs is largely asymptomatic and shedding can be increased before slaughter as pigs experience stress during, for example, mixing to create a slaughter batch, transport, handling and lairage in an unfamiliar environment [14]. This, together with the challenges of controlling *Salmonella* infection on farm, helps to explain the prevalence figures in caecal contents reported above. In this study, *Salmonella* prevalence in carcass swabs collected from the same animals was not investigated. Carcass swabs provide an indication of the residual *Salmonella* contamination on the pig carcass after the slaughter process, and better represent a proxy for the risk to public health [15]. It has been estimated that ~70% of the carcass contamination originates from the pig itself (after evisceration), whilst ~30% originates from cross-contamination [16]. Slaughter hygiene practices contribute significantly to the prevalence of pork carcass contamination, both from evisceration and environmental contamination, and a significant difference between *Salmonella* prevalence in caecal contents and in carcass swabs is therefore to be expected [17]. This was the case in the latest UK prevalence survey for pigs at slaughter, which reported a caecal contents sample prevalence of 30.5% and a carcass swab prevalence of 9.6% [3].

*S*. 4,[5],12:i:- and *S.* Derby were the most commonly isolated *Salmonella* serovars from caecal contents of UK pigs at slaughter in this study. *S*. Typhimurium (including monophasic variant) were the most frequently isolated *Salmonella* serovars from scanning surveillance in 2019. *S*. Derby was less commonly isolated, being the seventh most commonly isolated serovar from UK pigs [18].

*S*. Typhimurium (including monophasic variants) are of great importance for human health, and the transmission of these strains through the pork food chain is well documented (for example [19]). Combined, these serovars accounted for 41.1% of *Salmonella* isolated from the caecal contents in this study. Compared to the 2013 survey this represented a significant reduction in the proportion of positive isolates in each study (2013: 53.4%, 2019: 41.1%, *χ*^2^ test *P* = 0.038), and is partially due to the lower level of *S*. Typhimurium isolated in the recent study (2019: 4.5%, 2013: 19.0%) [3].

*S.* Derby is rarely involved in human disease in UK, but is more common in the EU [20]. It has consistently been the second most commonly reported *Salmonella* serovar isolated from caecal samples of UK pigs at slaughter. In the current study, *S.* Derby accounted for 25.9% of *Salmonella* isolated from UK pigs; an increase from 14.3% of positive samples in 2013 [3].

In a recent survey conducted in the UK on pork mince available at retail, *Salmonella* was detected from 5/342 (1.5%) of pork mince samples. Four of these were identified as S. Typhimurium (1.2%) and one as S. Derby (0.3%) [21]. Although not all of these samples originated from pigs reared in the UK, these figures highlight that S. Typhimurium and S. Derby detected from pigs at slaughter might contaminate retail meat at low levels.

A month by month variation of the *Salmonella* prevalence was observed in this study. Seasonal variation of *Salmonella* prevalence has been observed in other studies (for example [22] and [23]), but in these studies the variation was related to the increase in temperature during the summer months. Previous abattoir surveys conducted in GB and UK did not observe this variation, although one did not test samples collected over a 12 months period [3]. It is considered that the result may have been an artifact of the study design, with a chance occurrence of a greater proportion of positive herds being sampled in some months and not others.

Abattoir surveillance provides a means of assessing progress interventions along the food chain, up to the point of slaughter [24]. The GB pig industry has tried to introduce measures to control *Salmonella* in pig herds, through the implementation of controls such as increased biosecurity, improved cleaning and disinfection, use of organic acids and vaccination [25–27]. However, no significant difference in caecal sample prevalence was observed in comparison to the previous abattoir-based survey. Although comparable sampling and testing methods were used, it is expected that the proportion of carcass swabs reported to be contaminated with *Salmonella* remains low as detected in the previous study. This conclusion is supported by the fact that only 1.72% (95% CI 1.33–2.18%) of carcass swabs in the UK were contaminated with *Salmonella* in 2019 [1]. Continuing *Salmonella* surveillance at slaughter is recommended to assess the effect of farm and abattoir-based interventions and to monitor potential public health risks associated with consumption of *Salmonella-*contaminated pork products. This may be particularly important in demonstrating an effect of *Salmonella* vaccination if that becomes more widespread within the industry.

## Data Availability

The data described in this work is available in tables and figures.
